# Molecular Epidemiology of *Coxiella burnetii* from Ruminants in Q Fever Outbreak, the Netherlands

**DOI:** 10.3201/eid1704.101562

**Published:** 2011-04

**Authors:** Hendrik I.J. Roest, Robin C. Ruuls, Jeroen J.H.C. Tilburg, Marrigje H. Nabuurs-Franssen, Corné H.W. Klaassen, Piet Vellema, René van den Brom, Daan Dercksen, Willem Wouda, Marcel A.H. Spierenburg, Arco N. van der Spek, Rob Buijs, Albert G. de Boer, Peter Th.J. Willemsen, Fred G. van Zijderveld

**Affiliations:** Author affiliations: Central Veterinary Institute, part of Wageningen UR, Lelystad, the Netherlands (H.I.J. Roest, R.C. Ruuls, R. Buijs, A.G. de Boer, P.Th.J. Willemsen, F.G. van Zijderveld);; Canisius Wilhelmina Hospital, Nijmegen, the Netherlands (J.J.H.C. Tilburg, M.H. Nabuurs-Franssen, C.H.W. Klaassen);; Radboud University Medical Center, Nijmegen (M.H. Nabuurs-Franssen);; Animal Health Service, Deventer, the Netherlands (P. Vellema, R. van den Brom, D. Dercksen, W. Wouda);; Food and Consumer Product Safety Authority, The Hague, the Netherlands (M.A.H. Spierenburg, A.N. van der Spek)

**Keywords:** Q fever, Coxiella burnetii, molecular epidemiology, bacteria, zoonoses, goat, sheep, cattle, the Netherlands, research

## Abstract

Q fever is a zoonosis caused by the bacterium *Coxiella burnetii*. One of the largest reported outbreaks of Q fever in humans occurred in the Netherlands starting in 2007; epidemiologic investigations identified small ruminants as the source. To determine the genetic background of *C. burnetii* in domestic ruminants responsible for the human Q fever outbreak, we genotyped 126 *C. burnetii*–positive samples from ruminants by using a 10-loci multilocus variable-number tandem-repeat analyses panel and compared them with internationally known genotypes. One unique genotype predominated in dairy goat herds and 1 sheep herd in the human Q fever outbreak area in the south of the Netherlands. On the basis of 4 loci, this genotype is similar to a human genotype from the Netherlands. This finding strengthens the probability that this genotype of *C. burnetii* is responsible for the human Q fever epidemic in the Netherlands.

Q fever is a zoonosis caused by *Coxiella burnetii*, an intracellular gram-negative bacterium that is prevalent throughout the world (*1*). Domestic ruminants are considered the main reservoir for Q fever in humans (*2*). However, other animal species, including pet animals, birds, and several species of arthropods, can be infected by *C. burnetii* and cause human cases of Q fever (*2**–**5*). The main clinical manifestations of Q fever in goats and sheep are abortion and stillbirth. In cattle, Q fever has been associated with sporadic abortion, subfertility, and metritis (*4**,**6*). With an abortion, up to 1 billion *C. burnetii* per gram of placenta can be excreted (*7*). Most animal species that carry *C. burnetii* show no symptoms (*4*). Transmission to humans occurs mainly through inhalation of contaminated aerosols (*4**,**5**,**8**–**10*).

Recently, 2 DNA-based methods for typing *C. burnetii* were reported (*11**–**13*). Multispacer sequence typing is based on DNA sequence variations in 10 short intergenic regions and can be performed on isolated *C. burnetii* strains or directly on extracted DNA from clinical samples (*12**,**14**,**15*). Multilocus variable-number tandem-repeat analyses (MLVA) is based on variation in repeat number in tandemly repeated DNA elements on multiple loci in the genome of *C. burnetii* and might be more discriminatory than multispacer sequence typing (*13**,**15*). MLVA also can be performed on *C. burnetii* strains (*11**,**15*) or directly on DNA extracted from clinical samples (*16*). A total of 17 different minisatellite and microsatellite repeat markers have been described (*11*).

Starting in 2007, the Netherlands has been confronted with one of the largest Q fever outbreaks in the world, involving 3,921 human cases in 4 successive years. On 28 dairy goat farms and 2 dairy sheep farms, abortion storms (with abortion rates up to 80%) caused by Q fever were diagnosed during 2005–2009. These small ruminants are considered the source of the human Q fever outbreak in the Netherlands (*17*). The connection between Q fever abortion storms in small ruminants and human Q fever cases is based primarily on epidemiologic investigations (*18**–**21*). A limited investigation by genotyping with MLVA recently showed that farms and humans in the Netherlands are infected by multiple different, yet closely related, genotypes of *C. burnetii* (*16*).

Although dairy goats and dairy sheep appear to be the source of the human Q fever outbreak in the Netherlands, no information is available about the genetic background of *C. burnetii* in these populations. This knowledge is essential for gaining insight into the molecular epidemiology of the organism and the origin of the outbreak, as well as for outbreak management purposes.

Our objective was to show the genetic background of *C. burnetii* in domestic ruminants responsible for the human Q fever outbreak. This information is necessary to evaluate the epidemiologic link between the source and human cases and to compare the outbreak genotypes with internationally known genotypes. During 2008–2010, a total of 125 *C. burnetii*–positive samples from 14 dairy goat farms, 1 dairy cattle farm, and 2 sheep farms were typed by MLVA. In addition, we show the geographic distribution of these *C. burnetii* genotypes across the Netherlands and compare the genotypes with what is internationally known.

## Materials and Methods

### Animal Samples

**Figure 1:**
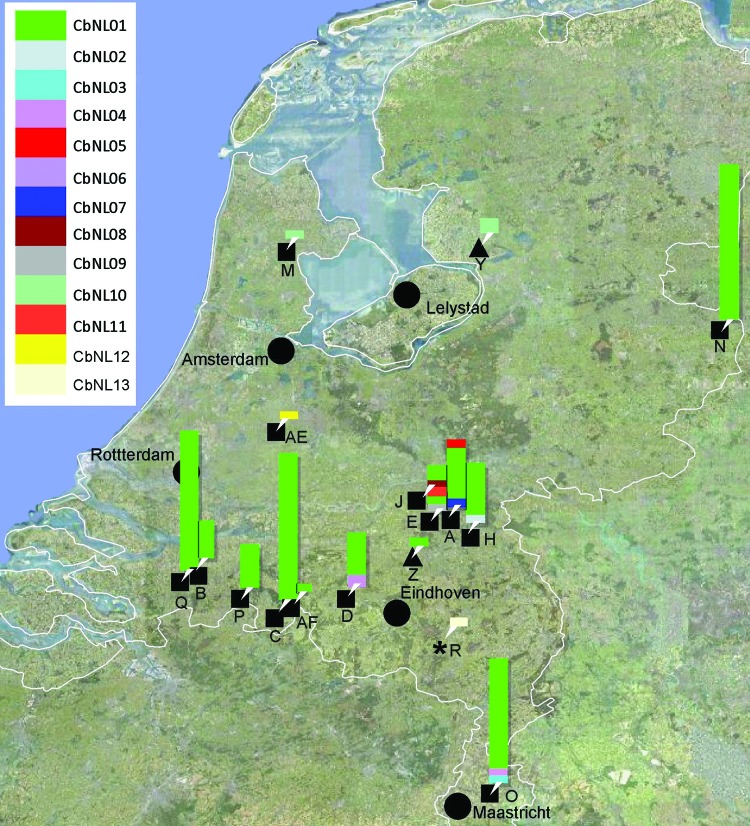
Map of the Netherlands showing locations of farms sampled during the Q fever outbreak, 2007–2010. Farms are indicated by letter and ruminant species (black squares, goats; black triangles, sheep; black star, cattle); genotypes of *Coxiella burnetii* found per farm are indicated by bars at each farm’s location. The height of the bar indicates numbers of isolates per genotype.

Our study comprised 14 dairy goat farms (farms A–E, H, J, M, N, O, P, Q, AE, and AF), 1 dairy cattle farm (farm R), and 2 sheep farms (1 dairy sheep farm Y and 1 sheep farm Z) sampled during the Q fever outbreak in the Netherlands (Table 1; Figure 1). On 12 of the 14 dairy goat farms, multiple abortions had occurred. On 2 dairy goat farms (farms J and M) and on the dairy sheep farm (farm Y), no abortions had occurred. On 1 dairy cattle farm and on the sheep farm (farm Z), *C. burnetii* was detected in a placenta after abortion. One goat farm (farm AG) sampled in 2001 was included with an archived histologic section of paraffin-embedded placenta from an abortion outbreak caused by *C. burnetii* infection.

**Table 1:** Overview of *Coxiella burnetii* genotyping results for farms sampled during human Q fever outbreak, the Netherlands, 2007–2010*

Farm ID	Animal species	Approximate herd size	Year of sampling	Approximate abortions in year of sampling, %	Sample type	No. samples tested	No. samples included in study	MLVA typing results	
								MLVA ID	No. samples
A	Dairy goats	617	2008	25	Vaginal swabs	20	9	CbNL01	7
								CbNL05	1
								CbNL07	1
B	Dairy goats	598	2008	20	Vaginal swabs	20	5	CbNL01	5
C	Dairy goats	546	2008	25	Vaginal swabs	20	20	CbNL01	20
D	Dairy goats	1,498	2008	19	Vaginal swabs	39	7	CbNL01	6
								CbNL04	1
E	Dairy goats	1,568	2008	8 (2007)	Fetal tissue	3	3	CbNL01	1
								CbNL09	1
								CbNL11	1
H	Dairy goats	606	2008	80	Vaginal swabs	13	8	CbNL01	7
								CbNL02	1
J	Dairy goats	459	2008	None	Vaginal swabs	3	3	CbNL01	2
								CbNL08	1
M	Dairy goats	769	2008	None	Vaginal swabs	2	1	CbNL10	1
N	Dairy goats	1,187	2009	25	Vaginal swabs	20	20	CbNL01	20
					Placenta	1	1	CbNL01	1
O	Dairy goats	83	2009	7	Vaginal swabs	40	16	CbNL01	14
								CbNL03	1
								CbNL06	1
					Milk	1	1	CbNL01	1
P	Dairy goats	548	2009	10	Vaginal swabs	20	6	CbNL01	6
Q	Dairy goats	340	2009	10	Vaginal swabs	25	19	CbNL01	19
AE	Dairy goats	500	2007	>5	Placenta	1	1	CbNL12	1
AF	Dairy goats	2,000	2007	>5	Placenta	1	1	CbNL01	1
AG	Dairy goats	590	2001	>5	Paraffin-embedded placenta	1	1		1
R	Dairy cattle	70	2007	<5	Placenta	1	1	CbNL13	1
Y	Dairy sheep	184	2010	None	Vaginal swabs	5	1	CbNL10	1
					Bulk tank milk sample	1	1	CbNL10	1
Z	Sheep	2	2009	50	Placenta	1	1	CbNL01	1

*ID, identification; MLVA, multilocus variable-number tandem-repeat analysis.

Vaginal swabs and milk samples from dairy goats and dairy sheep were sent to the national reference laboratory for notifiable animal diseases (the Central Veterinary Institute, part of Wageningen UR) by the Dutch Food and Consumer Product Safety Authority in accordance with the regulation in place at that time. These samples were submitted for confirmation testing of farms with clinically suspected Q fever (farms A–D, N, O, P, and Q), for tracing the source of human Q fever cases (because of proximity to human case-patients, farms H, J, and M) or for bulk tank milk monitoring (farm Y). Samples of immunohistochemically confirmed Q fever–positive goat and sheep placentas (farms N, AE, AF, and Z) and fetal tissue (farm E) were provided by the Animal Health Service, including 1 archived histologic section of paraffin-embedded placenta from a *C. burnetii* abortion outbreak in a goat farm in 2001 (farm AG), which was diagnosed retrospectively (*22*). The sampled dairy goat farms represent 60% of the farms with known abortion problems during 2007–2009.

### Testing of Samples before MLVA Typing

DNA was extracted from vaginal swabs and milk by using Chelex resin (InstaGene; Bio-Rad, Hercules, CA, USA). A vaginal swab tip or 200 μL of milk was added to 400 μL of Chelex suspension and incubated and shaken for 30 min at 56°C, followed by an incubation step for 8 min at 100°C. The clarified supernatant was used for PCR and MLVA. DNA from placentas was extracted by using a DNA tissue kit (DNeasy Blood and Tissue Kit; QIAGEN, Hilden, Germany). DNA from the paraffin-embedded placenta was extracted by using MagneSil Genomic Fixed Tissue System (Promega, Madison, WI, USA).

All samples were tested by an in-house real-time PCR directed toward the *C. burnetii*–specific IS*1111a* element (*23*). An inhibition control was constructed by using the primes of the IS*1111a* element (Table 2). PCR was performed on a 7500 Fast Real Time PCR system (Applied Biosystems, Foster City, CA, USA) by using 400 nmol/L of primers and 200 nmol/L of probes in 7 μL PerfeCTa Multiplex qPCR Supermix, uracil-n-glycosylase (2×) (New England Biolabs, Ipswich, MA, USA] with Low Rox dye (Quanta BioSciences, Gaithersburg, MD, USA ), 1 μL of inhibition control, 5 μL of sample, and 7 μL of water. An initial uracil DNA glycosylase (UDG) incubation for 5 min at 45°C and denaturation/activation for 60 s at 95°C was followed by 40 cycles of denaturation for 10 s at 95°C, annealing for 30 s at 60°C. Results were generated with 7500 Fast System Software (Applied Biosystems).

**Table 2:** Primers and probes used in the PCR for detecting *Coxiella burnetii* in clinical samples and loci and primers for MLVA of *C. burnetii*, the Netherlands, 2007–2010*

Identification	Temp, °C	Primer sequence for MLVA, with label indicated, 5′ → 3′	
		Forward	Reverse
Primers IS*1111a*	60	CATCACATTGCCGCGTTTAC	GGTTGGTCCCTCGACAACAT
Probe IS*1111a*	60	AATCCCCAACAACACCTCCTTATTCCCAC	
Probe inhibition control	60	ACATAATCTCTCCGACCCCACACTTCCATAC	
Cbu0448_ms03_12bp_7U_229bp	60	6-FAM-TTGTCGATAAATCGGGAAACTT	CACTGGGAAAAGGAGAAAAAGA
Cbu1963_ms21_12bp_6U_210bp	60	NED-AGCATCTGCCTTCTCAAGTTTC	TGGGAGGTAGAAGAAAAGATGG
Cbu1980_ms22_11bp_6U_246bp	60	PET-GGGGTTTGAACATAGCAATACC	CAATATCTCTTTCTCCCGCATT
Cbu0259_ms24_7bp_27U_344bp	65	VIC-ATGAAGAAAGGATGGAGGGACT	GATAGCCTGGACAGAGGACAGT
Cbu0838_ms27_6bp_4U_320bp†	65	6-FAM-GGGTCAGGTGGCGGGTGTG	TTCTCGCAAACGTCGCACTAACTC
Cbu0839_ms28_6bp_6U_480bp†	60	VIC-TAGAAACCGATAATCCCCTTGACA	ATTCCGCCGCCATTGAG
Cbu1351_ms30_18bp_6U_306bp‡	60	NED-ATTTCCTCGACATCAACGTCTT	AGTCGATTTGGAAACGGATAAA
Cbu1418_ms31_7bp_5U_285bp‡	60	PET-GGGCATCTAATCGAGATAATGG	TTTGAGAAAATTTTGGGTGCTT
Cbu1471_ms34_6bp_5U_210bp	60	6-FAM-TGACTATCAGCGACTCGAAGAA	TCGTGCGTTAGTGTGCTTATCT
Cbu1941_ms36_9bp_4U_477bp‡	65	VIC-GAAACCAGTCTTCCCTCAACAG	ATAACCGTCATCGTCACCTTCT

*MLVA, multilocus variable-number tandem-repeat analyses; temp, annealing temperature. †Different primer set from the proposed set by Arricau-Bouvery et al. (*11*). ‡Updated after personal communication with Le Flèche, Université Paris-Sud, Orsay Cedex, France.

### MLVA Typing

MLVA typing was performed by using a selection of 10 of the 17 loci described by Arricau-Bouvery et al. (*11*) according to the Multiple Loci VNTR Analysis databases for genotyping (http://minisatellites.u-psud.fr/MLVAnet/querypub1.php), except that Ms12 was omitted because of poor performance, and Ms24 was added (Table 2). New primers were designed for Ms27 and Ms28 to improve performance. The annotation of Ms30, Ms31, and Ms36 was updated (P. Le Flèche, pers. comm.).

The PCR amplification was performed by using an Applied Biosystems 9700 thermocycler in a total volume of 25 µL containing 1× reaction buffer, 1 U True Start *Taq* DNA polymerase (Fermentas, Glen Burnie, MD, USA), 2 mmol/L MgCl_2_, 0.2 mmol/L of each nucleotide (dATP, dGTP, dCTP, dUTP), 0.5 µmol/L of each primer, 0.5 U UDG (New England Biolabs), and 2–5 µL template. An initial UDG incubation for 5 min at 37°C and denaturation/activation for 2 min at 95°C was followed by 40 cycles of denaturation for 30 s at 95°C, annealing for 30 s at 60/65°C, elongation for 30 s at 72°C, followed by a final extension step for 5 min at 72°C. After the amplification, 0.5 U UDG inhibitor (New England Biolabs) was added to the PCRs to prevent further UDG activity. Up to 4 different PCR products with different fluorescent dyes were diluted, depending on the PCR efficiency, and pooled. From these pooled PCR products, 4 µL was mixed with 15 µL of Hi-Di formamide (Applied Biosystems) and 0.5 µL of GeneScan 600 LIZ Size Standard (Applied Biosystems). After denaturation for 3 min at 96°C the samples were cooled on ice. The PCR products were separated on a 3130 Genetic Analyzer (Applied Biosystems) with a 36-cm array by using POP7 polymer.

The fragments were sized by using GeneMapper version 4.0 software (Applied Biosystems). The accuracy of the sizing obtained by capillary electrophoresis was determined by comparing sequencing data from the reference strain with the obtained fragment size from the capillary electrophoresis and corrected if necessary. The number of repeats for each locus was determined on the basis of the published and corrected annotation of the various loci (Table 2). Non–whole repeat numbers were rounded off mathematically. Reproducibility was checked with positive controls.

### Data Analysis

The reference strain Nine Mile was used as reference (*11*). Analyses were performed, including only genotypes of *C. burnetii* containing <2 loci with missing values. Numerical typing data were imported into BioNumerics v 6.1 (Applied Maths, Sint-Martens-Latem, Belgium) and analyzed with the multistate categorical similarity coefficient by using unweighted pair group method with arithmetic mean clustering. Missing values were imported as question marks. The genotypic diversity of the population under study was calculated by using the adapted Simpson index of diversity (Hunter-Gaston diversity index [HGDI]) (*11**,**24*).

Found MLVA patterns based on the number of repeats per locus were called MLVA types and identified as CbNLxx. We compared MLVA types with MLVA types in the publicly accessible Multiple Loci VNTR Analysis databases for genotyping: Coxiella2007 and Coxiella2009_Netherlands (access date 2001 Jan 11). The Nine Mile strain was used as reference.

## Results

The study comprised 122 samples from 15 dairy goat farms, 2 samples from 1 dairy sheep farm, and 1 sample each from 1 sheep farm and 1 dairy cattle farm were included in this study (Table 1). Of the farms sampled during the outbreak, 13 were situated in the southern part of the Netherlands; 3 dairy goat farms (farms M, N, and AE) and 1 dairy sheep farm (farm Y) were located outside this area (Figure 1). From the 238 Q fever PCR-positive samples from the farms in this study, 125 (53%) yielded a genotype with <2 missing values: 52 with a complete genotype, 48 with 1 missing value, and 25 with 2 missing values. 113 (47%) PCR-positive samples represented partial genotypes with 3–10 missing values. From the paraffin-embedded placenta (farm AG), only a partial genotype could be shown, with 6 repeats on Ms03 and 10 repeats on Ms34.

**Figure 2:**
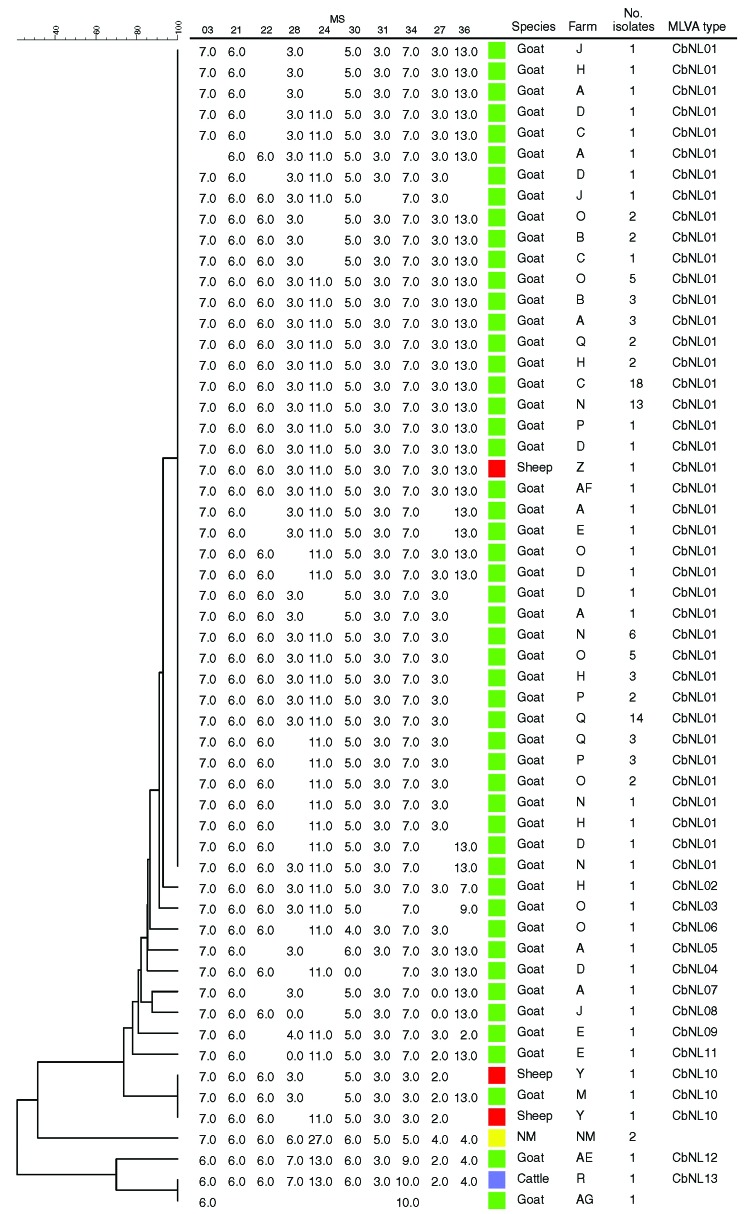
Phylogenetic tree with genotypes of *Coxiella burnetii* of all samples in the study, the Netherlands, on the basis of 10 multilocus variable-number tandem-repeat analyses (MLVA). Repeats per locus are shown; open spots indicate missing values. NM, Nine Mile reference strain.

We distinguished 13 genotypes in the 125 samples (CbNL01–CbNL13; Table 1; Figures 1, 2). All *C. burnetii* genotypes could be associated with abortion, except for 2 (CbNL10, farm M and Y; and CbNL08, farm J; Figure 1). The relationship between the genotypes in all samples is shown in Figure 2, including the genotype of the reference strain Nine Mile and the reference genotype of the reference strain Nine Mile from Arricau-Bouvery et al. (*11*), which were identical.

**Figure 3:**
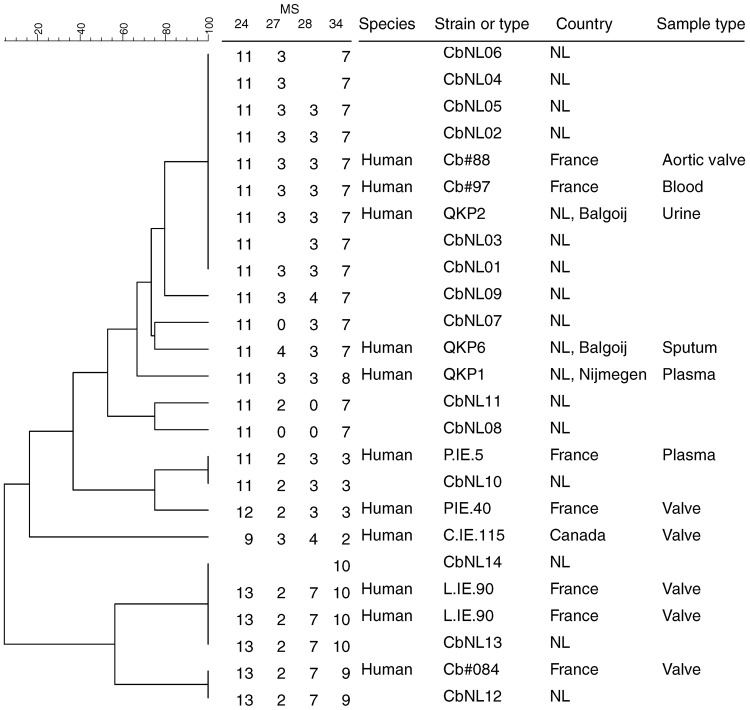
Phylogenetic tree with genotypes of *Coxiella burnetii* that are most closely related to the Dutch genotypes on the basis of 4 multilocus variable-number tandem-repeat analyses (MLVA). Genotypes are derived from the Multiple Loci VNTR Analysis databases for genotyping (http://minisatellites.u-psud.fr/MLVAnet/querypub1.php: Coxiella2009_Netherlands [accessed 2011 Jan 11]). Repeats per locus are shown; open spots indicate missing values. NL, the Netherlands.

**Figure 4:**
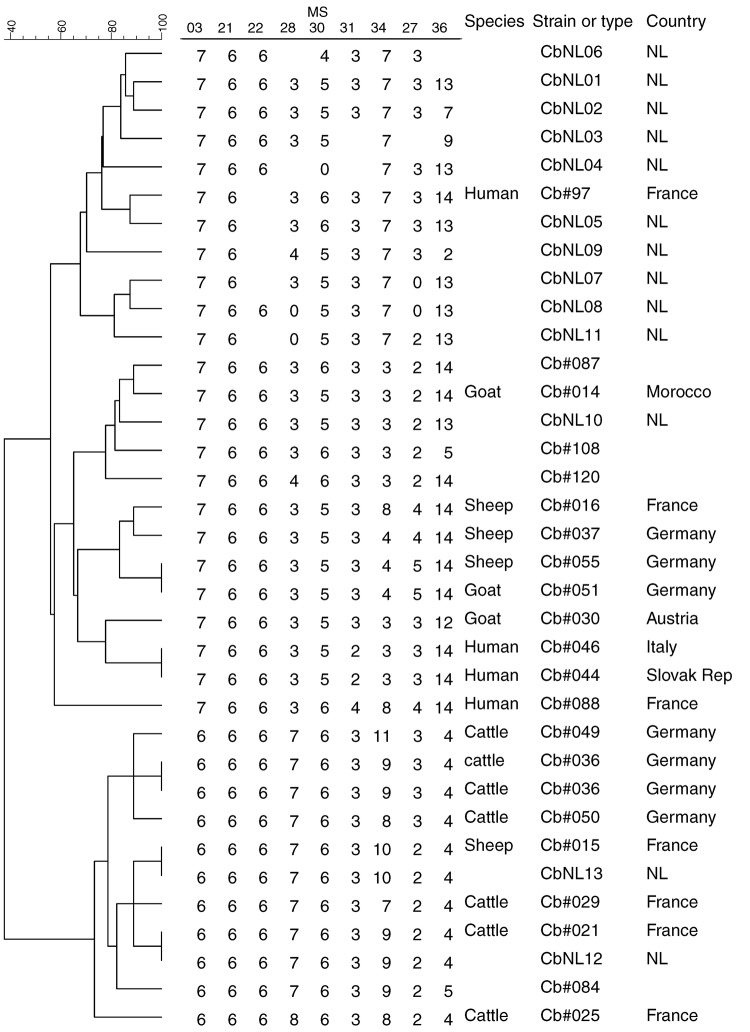
Phylogenetic tree with genotypes of *Coxiella burnetii* that are most closely related to the Dutch genotypes on the basis of 9 multilocus variable-number tandem-repeat analyses (MLVA). Genotypes are derived from the Multiple Loci VNTR Analysis databases for genotyping (http://minisatellites.u-psud.fr/MLVAnet/querypub1.php: Coxiella2007 [accessed 2011 Jan 11]). Repeats per locus are shown; open spots indicate missing values. NL, the Netherlands; Slovak Rep, Slovak Republic.

The 13 genotypes are separated in 2 clusters (Figure 2). One cluster containing a genotype represented by 111 (90%) of the samples (CbNL01); 1 genotype (CbNL10) represented by 3 samples (1 from a dairy goat farm and 2 from a dairy sheep farm); and 10 genotypes (CbNL02–CbNL09 and CbNL11) represented by 1 sample, all from dairy goat farms. The second cluster was distinctly separated from the other cluster, representing 2 genotypes in 1 dairy goat sample (CbNL12), in 1 dairy cattle sample (CbNL13) and the paraffin-embedded placenta. In samples from dairy goat farms with abortion problems, the same genotype (CbNL01) was present in 110 (91%) of 121 samples. One sheep sample also showed this genotype (farm Z). The geographic distribution of the genotypes according to the location of the originating farm is given in Figure 1. The relationship between the genotypes found in this study and the internationally known genotypes are presented in the phylogenetic trees in Figure 3 on the basis of 4 loci and in Figure 4 on the basis of 9 loci.

## Discussion

We performed MLVA typing of *C. burnetii* based on 10 loci on a large number of Q fever–positive samples to show the genetic background of *C. burnetii* in the domestic ruminants associated with the Q fever outbreak in humans in the Netherlands. In 125 (53%) of 237 samples, an adequate genotype for *C. burnetii* was generated. Previously, MLVA typing was performed on *C. burnetii* strains after primary isolation and cultivation (*11**,**13**,**15*) or, in the Netherlands, on only 11 clinical samples from humans, sheep, and goats with a selected number of 3 loci (*16*).

The main drawback of typing on clinical samples is the variable quality and amount of DNA. These drawbacks influence the typability of samples, resulting in partial genotypes; whether the missing values are caused by insufficient DNA concentrations and quality or by an absence of loci is unclear. If loci are absent, partial genotypes also are expected to be found in samples with high DNA loads. Such is not the case in our study. Typing of placenta material that contains high quantities of *C. burnetii*, as well as vaginal swabs with PCR cycle threshold (C_t_) values <32, yielded complete genotypes. In samples with C_t_ values of 32–34, only partial genotypes were obtained. Samples with a C_t_ value >34 were poorly typable.

Arricau-Bouvery et al. (*11*) calculated diversity indices for the 17 loci used in the MLVA, which varied from 0.28 for locus Ms22 to 0.86 for locus Ms34. The HGDI for the combined panels 1 and 2 of the MLVA typing method for *C. burnetii* can be calculated on 0.99 and for panel 2 on 0.92. These HGDIs are in the upper part of the 0.438–0.997 range reported by Hunter and Gaston (*24*) for typing methods for various bacteria and yeasts.

The high diversity indices for the MLVA of *C. burnetii* indicate a high discriminating power, and this capability makes MLVA typing suitable for distinguishing *C. burnetii* isolates. With this highly discriminatory typing method, we found that 1 genotype of *C. burnetii* predominated on all dairy goat farms in the southern part of the Netherlands. On 12 of 14 dairy goat farms, this genotype was found in 91% of samples, varying per farm from 33% (farm E) to 100% (farms B, C, N–Q, Table 1, Figures 1 and 2). Although the sample size was small compared with the number of animals on the farm (Table 1), these data show that 1 genotype was far more common than other genotypes found on these farms. The 9 other genotypes occurred once, each representing only 0.8% of all found genotypes on dairy goat farms. The most predominant genotype was found on all 11 dairy goat farms in the southern Netherlands and on a farm in the eastern part of the country (farm N). This finding strongly suggests a clonal spread of *C. burnetii* with this predominant genotype over the dairy goat farms in the southeastern part of the Netherlands.

The clonal spread of 1 genotype of *C. burnetii* could be explained by 2 phenomena. First, the dairy goat industry in the Netherlands sharply increased from almost 100,000 dairy goats in 2000 to >230,000 dairy goats on ≈350 farms in 2009 (*17*). Most of these goats were bred in the Netherlands, which probably resulted in a microbial relationship between many of the dairy goat herds. In this theory, the *C. burnetii* strain with the most predominant genotype was present in the Netherlands for a long period before the abortion problems in dairy goats started in 2005. This theory is not supported by the results of the typing of the paraffin-embedded placenta from an aborted dairy goat who in 2001. The typing result differs on 2 loci from the most predominant genotype found in this study. Second, clonal spread could have been facilitated by emergence of a genotype of *C. burnetii* causing abortion in dairy goats that could then spread successfully over the dense goat population in the southeastern part of the country. Whether this genotype is more virulent is subject to research.

On the basis of comparison of MLVA types on 4 loci (Figure 3), CbNL01–06 could not be distinguished and were similar to the genotype of a person in the Netherlands (QPK2) and 2 genotypes from persons in France (Cb#88, Cb#97). The sample from a person in the Netherlands is derived from patient 2 reported by Klaassen et al. (*16*). Patient 2 is the farmer of farm A, where genotype CbNL01 predominated, as well as CbNL05 (Table 1). This shows a genetic link between the *C. burnetii* DNA from the farmer and his abortive goats, which suggests that the farmer was infected by his own goats. However, this link is based on only 4 loci on 1 human sample. To further confirm the link between dairy goats and humans, more samples need to be typed with more MLVA loci to increase the discriminatory power.

The human sample with ID QKP6 is the same sample as that from patient 4 reported by Klaassen et al. (*16*) and is most closely related to CbNL07. Human sample QKP1 is the same as that of patient 1. Patient 5 fits in the genotype cluster in the Netherlands, as does patient 2. The sheep reported by Klaassen et al. did not abort, and their samples show a difference of 1 repeat on Ms34 compared with CbNL01. On the basis of the comparison of MLVA types on 9 loci (Figure 4), all genotypes in this study can be distinguished. The most predominant genotype CbNL01 clusters with other genotypes (CbNL02–CbNL09, CbNL11) and with 1 human sample (Cb#97) from France. CbNL01 differed from this human isolate on 2 loci (Ms30 and 36), which shows that the most predominant genotype in the Netherlands is unique. Whether this finding can be attributed to the small number of strains and clinical samples typed or is really a unique genotype is not yet clear. The closest relation to an isolate from France might give a clue about the origin of the genotype from the Netherlands.

The human Q fever outbreak in the Netherlands started in the southern part of the country and resulted in >3,500 human cases during 2007–2010. Dairy goats and dairy sheep are considered to be the source of this outbreak, primarily on the basis of epidemiologic findings (*10**,**17**,**20**,**21**,**25**,**26*). In our study, samples were typed from farms suspected of being the source of the human Q fever outbreak. Results show that 1 genotype of *C. burnetii* predominated in the dairy goats and sheep in the human Q fever outbreak area in the southern part of the Netherlands, and this genotype also was present in a human case-patient in the Netherlands. This *C. burnetii* genotype is expected to have played a key role in the Q fever outbreak in small ruminants in the Netherlands and was also transmitted widely to humans, causing Q fever in the human population. If this hypothesis holds true, *C. burnetii* with the same genotype as in dairy goats should be found in most samples from human Q fever patients. To this end, a study was performed to show the genetic background of human *C. burnetii* isolates in the Netherlands by using a concordant MLVA typing method (J.J.H.C. Tilburg et al., unpub. data). Furthermore, the uniqueness of the predominant genotype of *C. burnetii* for the Netherlands can be part of the explanation why the magnitude of the Q fever outbreak in the Netherlands has never been seen elsewhere.
